# Surimi and Low-Salt Surimi Gelation: Key Components to Enhance the Physicochemical Properties of Gels

**DOI:** 10.3390/gels11020142

**Published:** 2025-02-17

**Authors:** Noman Walayat, María Blanch, Helena M. Moreno

**Affiliations:** 1 College of Tea Science and Tea Culture, Zhejiang Agriculture and Forestry University, Hangzhou 311300, China; 20232004@zafu.edu.cn; 2 Department Section of Galenic Pharmacy and Food Technology, Veterinary Faculty, Complutense University of Madrid, 28040 Madrid, Spain

**Keywords:** surimi, low salt, gelation challenges, physicochemical properties, technological additives

## Abstract

Surimi-based products are nutritionally valuable due to their essential amino acid composition, their content of high-quality proteins with excellent digestibility, and their low fat content. However, to achieve the desired texture, a significant amount of salt (1–3%) must be added, which could compromise their health benefits. This study provides an overview of surimi production, the gelation mechanism of myosin, and the most relevant gelation enhancers that could be used in manufacturing low-salt surimi-based products. Reducing the salt content in surimi-based products presents a significant challenge for the industry, not only from technological and sensory perspectives but also in response to the growing demand of consumers for healthier food options. So, this manuscript highlights several strategies for achieving optimal quality characteristics in relation to functional properties for the surimi products industry. In addition, surimi as a raw material is often misunderstood by consumers, who may question its nutritional value and, consequently, its consumption. Therefore, it is crucial to thoroughly explain the processing of this raw material and emphasize the importance of proper myofibrillar protein gelation to develop high-value surimi-based products.

## 1. Introduction

The nutritional quality of fishery products, especially those made from surimi, will depend mainly on the species from which the surimi is made and the ingredients added to the product. Surimi is mainly composed of water and myofibrillar proteins, so the higher the percentage of surimi used in the production of surimi products (kamaboko, chikuwa, fish balls, sausages, etc.), the higher the nutritional quality of the product. Surimi contains proteins of high biological value and good digestibility (due to the composition of essential amino acids) and has a low fat content [1]. In addition, its possible bioactive effects have been studied, and it was found that its protein could be effective in the control of dementia [2], could prevent colon cancer [3], or could inhibit the absorption of fat and sugar [4], among other benefits.

However, the preparation of surimi gels requires the addition of a significant amount of salt (1–3%) to achieve the desired texture, which may compromise their health benefits and, consequently, their consumption. As a result, the surimi products industry is working to reduce the salt content due to growing health concerns in surimi products by replacing it with alternative compounds, such as seaweed extract, potassium chloride, protein hydrolysates, and amino acids, or by employing different processes that enable the production of low-salt surimi-based products [5].

Common salt (NaCl) is the main source of sodium in the diet. Sodium is necessary for the proper functioning of the body; however, epidemiological studies worldwide suggest that salt consumption is an inducer of increased blood pressure [6]. As reported by the World Health Organization [7], arterial hypertension is one of the leading causes of premature death worldwide. For that reason, one of the global goals related to non-communicable diseases is to reduce the prevalence of hypertension by 25% by 2030 compared to the 2010 baseline levels.

To face the increasing concerns about sodium intake, various countries and regions have enacted sodium labelling laws to indicate the salt content in food products. These labels generally classify sodium levels as no sodium (<5 mg), very low sodium (<35 mg), low sodium (<140 mg), reduced sodium (<25%), and light sodium (<50%) [8]; however, the criteria for sodium labelling vary across countries. The European Union adopted “Regulation N° 1924/2006 (2006) of the European Parliament and of the Council on nutrition and health claims made on foods”, which applies to all nutrition and health claims made in commercial communications [9]. As a result, the food industry, particularly the surimi products industry, is facing a significant challenge in reducing the salt content of surimi gel products while maintaining their quality [10]. Although significant efforts have been made to develop innovative salt reduction processing methods, researchers have faced numerous challenges, such as compromised gel quality, increased costs, and safety concerns [11].

The global production of surimi has been estimated at approximately 800,000 tons in 2023. This volume reflects a robust global market, led by countries such as Japan, the United States, and Russia, which has shown an increasing interest in expanding its production capacity, exemplified by its plan to triple surimi production in the coming years. The industry is supported by strong demand due to the versatile use of surimi in processed food products and its popularity in gastronomy [12]. The social changes that have taken place in recent years have led to an increase in the consumption of fishery products and, more specifically, surimi-based products. This increase in the consumption of these products is mainly due to the fact that they have a competitive price compared to other fishery products [13,14].

In the processing of surimi-based products, the final texture is crucial, as these products are often analogues that must replicate the characteristic texture of the original product they aim to imitate. Approximately 20% to 25% of the world’s produced surimi is used in the manufacture of crab substitute [13,15]. To achieve an adequate texture in these products, a significant amount of salt (commonly 1–3% NaCl) is required to promote the thermal gelation of myofibrillar proteins [16,17,18]. As stated before, this fact makes it difficult to comply with nutritional requirements that call for a reduction in salt in the diet and seek to promote the consumption of healthy foods. Meanwhile, considering the role of salt in the techno-functional characteristics of surimi-based products, mainly texture, its reduction represents an important technological challenge for industry. According to this, different technological additives have been studied as potential enhancers of the gelation of low-salt surimi-based products [5,19,20,21,22,23]. Therefore, the objective of this work is to provide an in-depth descriptions of surimi as a raw material and the process of surimi gel formation, as well as to explore various alternatives that the surimi products industry can employ to compensate for the reduction in salt in terms of physicochemical properties, particularly texture. This includes optimizing the use of technological additives that promote surimi gelation without compromising the final physicochemical properties of the final products.

## 2. Surimi

The origin of the word “surimi” is Japanese and means “minced fish”. Basically, it refers to the frozen block of fish myofibrillar protein concentrate that is mixed with cryoprotectants and finally undergoes freezing. This product is sold to food processors, who blend it with other ingredients to impart texture, taste, and colour [24]. In Japan, surimi has traditionally been used to produce a product called kamaboko (a mildly flavoured fish gel that is nearly tasteless and widely used in Japanese cuisine). However, it is now also employed in the production of substitutes for high-value seafood products, aiming to replicate their texture, flavour, and appearance [25]. Additionally, particularly in Japan, but also in the United States and Europe, various surimi-based products, either alone or combined with other ingredients, are becoming increasingly popular as snacks, representing a growing sector in the food industry. Surimi commercialization was initially limited to Japan and some Asian countries, but its worldwide expansion took place in the 1960s, when the use of low-molecular-weight carbohydrates (sucrose and sorbitol) was optimized as efficient cryoprotectants of fish protein, facilitating its commercialization in the freezing stage [15].

Surimi was mainly made from Alaska pollock (*Theragra chalcogramma*), a very abundant but undervalued fish due to its soft texture. However, the surimi obtained from this fish species has excellent gelling properties and is the base of the technological development of surimi-based products [15,26]. The increase in surimi production to meet consumers’ needs, the raw material costs, market fluctuations, and the use of new technologies have boosted its production from other species of greater availability and/or lower economic value, such as hake (*Merluccius gayi*; *Merluccius productus*), horse mackerel (*Trachurus trachurus*), sardine (Sardine sp.), Pacific halibut (*Atheresthes stomias*), southern blue whiting (*Micromesistius australis*), blue-tailed hake (*Macruronus novaezelandiae*), Japanese bogue (Nemimpterus sp.), etc. These species are underutilized in many parts of the world and have sufficient properties for the manufacture of quality surimi [14,27,28].

### 2.1. Surimi Processing

**Figure 1:**
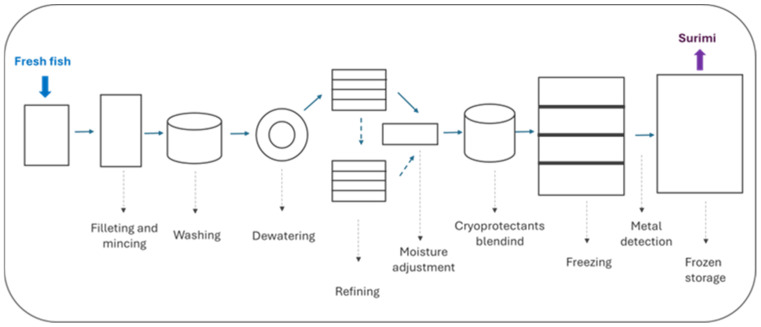
Flow chart of surimi manufacturing.

The surimi manufacturing process begins with the capture of the fish and ends with the storage of the surimi in a frozen state (Figure 1).

This process has been extensively described by several authors [15,25] and can be synthesized as follows: the fish is quickly beheaded and gutted to prevent intestinal enzymes from migrating to the muscle and damaging myofibrillar proteins. It is then washed thoroughly with water, followed by the removal of skin, bones, scraps, cartilage, and as many impurities as possible before being minced. The minced fish is washed several times with water or saline solutions (at temperatures between 5 and 10 °C) to remove soluble proteins, mainly sarcoplasmic proteins, and other impurities that could reduce the gelation capacity of the surimi. The next step is the refining process, which eliminates additional impurities before moisture adjustment (to around 80%). In addition to that, cryoprotectants are then incorporated to ensure that the proteins maintain maximum functionality after freezing, resulting in high-quality surimi. Commonly used cryoprotectants include sucrose and sorbitol (4% of each), while polyphosphates (0.3%) are also added to facilitate protein solubilization and enhance gel elasticity. Depending on the species, other additives may be used to adjust the pH, inhibit enzymes, and/or chelate metals, helping to preserve protein functionality. Surimi is typically frozen in contact plate freezers (to a thermal centre temperature of −20 °C) in standard 10 kg blocks. It is then packaged in cardboard boxes containing two 10 kg blocks of surimi. According to surimi processing, factors such as the solubility of myofibrillar proteins as affected by ionic strength, pH, washing cycles, the wash/meat (W/M) ratio, and proteolysis are important considerations taken into account by surimi processing industries before selecting the fish species used to make surimi.

### 2.2. Surimi Quality

The key factor that determines surimi quality and is closely linked to its freshness is the functionality of surimi myofibrillar protein [29]. This functionality primarily depends on whether processing was carried out properly and under optimal conditions, as well as the type of fish being used. 

There is not a standardized method for raw surimi quality evaluation. In the case of Alaska pollock (*Theragra chalcogramma*) surimi, its quality is assessed by its gel-forming ability (measured through texture tests) although gel properties change greatly depending on the cooking process especially preheating procedures for surimi gelation [15]. During surimi production, the use of large machinery (Figure 1) presents a significant risk of contamination or cross-contamination. One of the relevant hazards that might occur during raw surimi processing is the potential inclusion of metal fragments [15]. Nevertheless, current industrial processing includes a metal detector before frozen storage (Figure 1).

On the other hand, Surimi is the main ingredient in surimi seafood products, which are cooked and/or pasteurized during production, therefore the presence of microorganisms in raw surimi is more of a quality concern than a safety issue [15]. 

Surimi is usually classified according to the properties exhibited by Alaska pollock (*Theragra chalcogramma*) surimi. Its quality is assessed by its gel-forming ability (measured through texture tests), colour (the whiter the colour, the higher the quality), purity (the complete absence of blood, bits of skin, and dark meat indicates greater purity), homogeneity, and microbiological and enzymatic quality [24]. These characteristics result in a three-grade grading system: the primary grade (SA, FA, A), which refers to the surimi that comes from the first refining process; the secondary grade (KA), which comes from a second refining process; and the recovered grade (KB, RA), which refers to the surimi that has undergone two refining processes and comes from discards.

## 3. Surimi Gelation

Surimi-based products primarily rely on thermal gelation, which is influenced by several factors. Therefore, it is important to describe the factors that contribute to the proper gelation process.

### 3.1. Main Factors Affecting Gelation Process

#### 3.1.1. Protein Content

The gelling capacity of surimi is mainly attributed to the presence of myofibrillar proteins, especially myosin, that account for about 55–60% of the total protein content [17]. In the gelation phenomenon, myosin must be dissociated by the addition of salts, and then myosin heads predominantly aggregate through disulfide bonds, while the hydrophobic effect drives tail aggregation [30,31,32,33]. During the washing process in the production of surimi, the non-functional components present in the fish muscle (blood, pigments, impurities, and sarcoplasmic proteins, which are largely enzymes) are removed, and the functional myofibrillar proteins are concentrated, which enhances the gelation capacity of the surimi.

#### 3.1.2. Salt Content

The addition of salt, usually 1–3%, is necessary to solubilize and disperse the myofibrillar proteins [17,34]. In fact, gelation does not occur or does so defectively in the absence of salt because myofibrillar proteins are insoluble at a low ionic strength, and to facilitate the formation of a well-organized and stabilized protein network, the protein must be solubilized. At low salt concentrations (<0.3 mol/L), myosin tends to form a discontinuous network that is a porous structure [35], while high-salt conditions (≥0.3 mol/L) enable the formation of a three-dimensional network gel [17]. During the homogenization process with NaCl, the salt ions (Cl^−^ and Na^+^) bind to the oppositely charged groups on the protein surface, resulting in a disruption of the intermolecular ionic bonds of the myofibrillar proteins. This increases the affinity of proteins (mainly myosin) for water molecules and allows for their solubilization by partially unfolding the structure of the myosin molecule, favouring gelation [17,18].

#### 3.1.3. pH

Changes in pH can alter molecular interactions and the spatial conformations of proteins. When the pH is close to the isoelectric point, the proteins present zero net charge; therefore, the repulsion between proteins is lower and they tend to precipitate due to minimal solubility, giving rise to less hydrated and less firm gels [30]. When the pH is located above or below the isoelectric point, the proteins are negatively or positively charged so that the chains of the protein molecules repel each other and are therefore more susceptible to binding with the water molecules present in the medium, increasing the water retention capacity of the protein gels [22,25].

#### 3.1.4. Effect of Endogenous Transglutaminase (TGe)

The endogenous transglutaminase is an enzyme naturally found in fish muscle that catalyses the formation of non-disulfide covalent bonds (ε-(γ-glutamyl) lysine), resulting in the formation of myosin polymers, which is correlated with increased gel strength [16,36]. TGe activity is calcium ion-dependent, so it can be modulated depending on the presence of calcium ions in the medium. TGe acts at low temperatures (<40 °C); therefore, to favour its action, the gelation process starts with a “setting” period, which consists of starting with a 5–40 °C treatment for a certain period of time to obtain highly deformable gels called suwari gel [37,38].

### 3.2. Thermal Gelation of Myofibrillar Proteins

**Figure 2:**
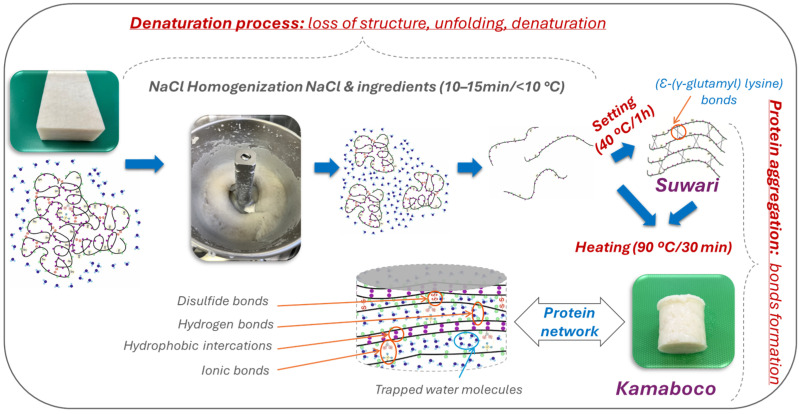
Denaturation or unfolding of proteins, followed by intermolecular aggregation of myofibrillar proteins during thermal gelation of surimi.

Focusing on the modification that occurs in proteins during the gelation process, these can be described in two phases: denaturation or unfolding of the proteins, followed by intermolecular aggregation of the proteins (Figure 2). The gel formation process begins with the mixing process of surimi and salt, which solubilizes the proteins, resulting in the formation of a viscous mass. The progressive increase in temperature results in the formation of a matrix that finally transforms into a gel with viscoelastic solid properties [22,39].

#### 3.2.1. Denaturation Process of Myofibrillar Proteins

The gelation process starts with the solubilization of the proteins by the addition of salt (commonly NaCl). During this process, myosin loses its quaternary, tertiary, and secondary structures, so the α-helix structure stabilized by non-covalent bonds, mainly hydrogen bonds, unfolds. The loss of this structure gives rise to the formation of a β-sheet structure stabilized by non-covalent bonds [16]. This denaturation results in the exposure of reactive groups, resulting in a high hydration of the proteins upon interaction with the water molecules present in the medium. These reactive groups will be involved in the formation of bonds of different types during the aggregation stage (Figure 2). Denaturation proceeds continuously and can occur to varying degrees, and during this process, a large number of hydrogen bonds are broken between the carboxyl and amino groups of the polypeptide chain of proteins responsible for keeping the native structure of the protein folded [17,40,41].

#### 3.2.2. Myofibrillar Protein Aggregation Process

Aggregation refers to the formation of protein–protein interactions that lead to the creation of large, high-molecular-weight protein complexes. Gelation is the process where previously unfolded proteins aggregate into a three-dimensional structure, with protein–protein and protein–solvent interactions forming an organized protein matrix that can retain a substantial amount of water molecules [42]. As the thermal denaturation temperature of proteins varies among different fish species, it is necessary to study the most appropriate time and temperature according to the specific properties of fish species [10].

During the initial phase of the heating process, myosin molecules undergo denaturation, which causes the helical structure of the tail to gradually unfold and reveal the previously hidden active groups. When the molecule is in its folded state, the activity of disulfide bonds is low; however, when proteins are unfolded by any agent, the reactivity and formation of these bonds increase [43]. Consequently, these proteins connect through intermolecular interactions. As the temperature continues to rise, hydrophobic interactions and disulfide bonds between proteins are strengthened, leading to the formation of an organized three-dimensional reticular gel structure [44,45] (Figure 2).

The main types of bonds involved in the gelation of myofibrillar proteins are hydrogen bonding, ionic interactions or salt bridges, hydrophobic interactions, and covalent bonds [16]. Ionic interactions are those formed between positive and negative charges on the protein surface. At a neutral pH, the carboxyl groups (COO^−^) are negatively charged, while the amino groups (NH_2_^+^) are positively charged. These groups attract each other, forming such interactions. The addition of salt to surimi breaks the ionic bonds and aids in the dispersion of myofibrillar proteins [41]. On the other hand, hydrophobic interactions, like disulfide bonds, are formed by the action of heat or high-pressure (>300 MPa) treatments [46]. The formation of these bonds also occurs as a consequence of the unfolding of the protein structure, which exposes the hydrophobic residues and reactive groups inside the protein molecule. The association of the hydrophobic zones decreases the entropy of the system, resulting in balanced bonding, which concludes with the formation of an ordered three-dimensional structure [17]. Finally, disulfide (S-S) bonds are formed by the oxidation of sulfhydryl groups present on cysteine residues. Their formation is considered to be thermo-irreversible [44,45] (Figure 2). Additionally, the addition of oxidants can accelerate the formation of intermolecular disulfide bonds [47,48].

The gelation process of surimi is significantly affected by the heating method and conditions applied. Research has shown that the breaking force achieved with two-stage heating (firstly at 40 °C/60 min and secondly at 90 °C/30 min) is considerably stronger than that obtained with a single heating treatment [49]. This is because a single high-temperature heating treatment (121 °C) leads to myosin degradation due to the disintegration of the protein’s secondary structure and the weakening of binding forces (hydrophobic interactions and ionic bonds) responsible for maintaining the stability of the gel. The resulting gel has a highly porous and discontinuous microstructure. In contrast, low-temperature pre-treatment (40 °C) assists TGe action, advocating the formation of covalent cross-linking bonds of ε-(γ-glutamyl) lysine. TGe efficiently binds protein molecules together, avoiding the thermal breakdown of myosin at high temperatures. The result is a dense three-dimensional network. For this reason, performing surimi gelation in two steps is the most convenient heating gelation method to obtain a firm and elastic surimi gel [20,50,51].

## 4. Using Gelation Enhancers as a Strategy for Surimi Gelation with a Low Salt Content

Salt plays a crucial role in the production of surimi-based products, making its reduction a significant challenge for the food industry in the manufacturing of these products.

### 4.1. Substitution of NaCl with Other Salts

This is the most used method; however, modifications in the presence of metal salt and in their amount can alter the forces and spatial conformation relating to myosin molecules, thereby affecting the properties of the resulting gel [52]. In particular, substitution with potassium chloride has certain disadvantages, such as adding strange flavours to the product; in fact, the substitution of 50% NaCl with 50% KCl has been described to significantly increase bitterness and to reduce the salt appreciation [53], although substitution with 25% KCl resulted in a gel with very appropriate physicochemical properties, which can be attributed to the great exposure of amino acid residues and hydrophobic groups in combination with the decrease in the alpha helix presence and the growth in the beta sheet presence of myofibrillar proteins that, at the end, enhance the water-holding capacity, maintaining the desired taste and improving the gel properties. Other salts, such as CaCl_2_ and MgCl_2_, have been tested without very positive results since the solubilization of myofibrillar proteins is facilitated, resulting in an organized and dense gel network with significant water-holding capacity [54]. Therefore, the type of salt and the amount added must be carefully tested in each case.

However, compared to salt, using the ingredients mentioned above could considerably increase the cost of the products, add extra calories, and reduce the nutritional value of the low-salt surimi gel.

### 4.2. Incorporation of Different Gelation Enhancers in Low-Salt Surimi Gels

The addition of small quantities of ingredients and/or adjuvants that improve or modify certain characteristics of foods is a common practice in the food industry. Therefore, many exogenous additives have been studied in the manufacture of surimi-based products and low-salt surimi-based products to improve the gel properties [22]. All of these ingredients serve a technological function; however, they also have several drawbacks from a nutritional standpoint. They could contribute extra empty calories to the final product and may contain allergens (such as milk protein and egg white), among other concerns.

#### 4.2.1. Addition of Microbial Transglutaminase

The use of microbial transglutaminase (MTGase) to enhance the texture of myobibrillar protein gels from both fish and meat has been extensively studied [23,55]. However, MTGase might diminish gel strength due to excessive cross-linking, which could reduce the interaction between proteins and water as it constantly enhances cross-linking among proteins [56]. This could result in water loss within the system, causing the gel to become overly firm and less flexible, ultimately leading to an inelastic and fragile gel [23,57]. So, the use of MTGase should be optimized according to the surimi fish species, gelation process, and the presence of other technological adjuvants or salts [20,55].

#### 4.2.2. Addition of Polyphenols

Polyphenols have been studied as gelation enhancers since they can function as protein cross-linkers. These compounds possess multiple hydroxyl groups that can trigger the formation of hydrogen bonds and interact with hydrophobic amino acids via their non-polar aromatic ring [58]. Additionally, phenols can oxidize to quinones, which may react with sulfhydryl and amino groups in the protein to create a covalent bond (C–S or C–N) [59]. Polyphenols from apple [60], tea [61], and olive leaf powder [62], among others, have demonstrated a positive effect on the gel strength of heat-induced surimi gels, although their effect on other physicochemical properties, such as colour and water-holding capacity, has to be considered, as well as the effect of the interaction with other technological adjuvants [23].

#### 4.2.3. Addition of Phosphates

Phosphates are commonly added to surimi as cryoprotectants, usually in the form of sodium tripolyphosphate or tetrasodium pyrophosphate. These compounds prevent actin and myosin binding and thus myosin aggregation in frozen storage, resulting in improved gelling ability in different surimi types [25,63,64]. However, although they are sodium salts and, in percentage terms, provide a sodium content similar to that of NaCl (31.24% compared to the 39.34% that NaCl would provide), their advantage lies in the fact that they are added in lower percentages than NaCl to achieve similar effects [65]. Sodium pyrophosphate can enhance the water-holding capacity and strength of surimi gel [63,66]. On the other hand, a correlation has been described between the effect of sodium phosphate and the pH of the medium, indicating that an increase in the phosphate levels at an alkaline pH in rainbow trout surimi resulted in better emulsification properties and poorer gelation ability [67]. A positive effect in enhancing surimi gelation in low-salt surimi gels has also been described for tetra-sodium pyrophosphate at very low concentrations (0.05%) [5]. However, although phosphate compounds have been proven as promising processing agents, they most likely have a detrimental effect on gel properties as they may chelate the Ca^2+^ ion. This could hinder the gelling of surimi induced by endogenous transglutaminase [63], which indicates the need to carefully study the range at which they are added to surimi.

#### 4.2.4. Addition of Hydrocolloids

Polysaccharides (e.g., agar gum, K-carrageenan, curdlan, and fucoidan) and some proteins, such as gelatine, are hydrocolloids that significantly impact the texture and functional characteristics of restructured and surimi gels [33,68,69,70].

Hydrocolloids can absorb water and expand during heating, which fills in the network of surimi gels and exerts pressure on the protein network, thus increasing surimi gel strength [33,71,72,73]. In addition, some hydrocolloids can form a thermo-irreversible gel once the temperature rises to 80 °C, enhancing the gel strength of surimi [74]. Recently, yeast β-glucan, konjac glucomannan, and deacetylated konjac glucomannan, as functional polysaccharides, have been used as additives to improve surimi gel properties [75]. In general, hydrocolloids can interact with myofibrillar proteins through non-covalent bonds and electrostatic interactions. These interactions help stabilize the gel network and improve its texture, enhancing both gel strength and elasticity. As a result, they form a more cohesive and stable gel matrix, which improves the overall mechanical properties. However, the best effect on the mechanical properties of low-salt surimi gels was observed when treatment such as high-pressure processing was applied to aid the gelation process [21].

On the other hand, the addition of a small amount of gelatine has been reported to increase surimi gel’s strength, which could be attributed to its water binding ability [76]. In addition, the effect of hydroxypropyl-methylcellulose (HPMC), a polysaccharide derived from cellulose able to form a thermo-reversible gel, has been checked as a gelation enhancer. Although it did not result in a well-built combinative gel, the rheological and textural properties of horse mackerel surimi gel were enhanced [77]. Nevertheless, the addition of a higher concentration of hydrocolloids might inhibit the cross-linking of surimi proteins and disrupt the formation of an organized matrix, leading to a reduction in gel strength due to the dilution effect of myofibrillar proteins in surimi because of hydrocolloid addition [73].

#### 4.2.5. Addition of Amino Acids

A few recent studies have stated the positive effect of some amino acids on surimi gelation because these amino acids are amphoteric above their isoelectric point [5,78]. They can supply ionic strength to the medium, which is particularly interesting because, under low-salt conditions, myosin remains as insoluble fibres that are difficult to fully solubilize and disperse [79]. However, the charged amino acids in the dissolution of myofibrillar protein may promote the orderly interaction of protein molecules during heating. Firstly, basic amino acids such as Arg, Lys, and His can cooperate electrostatically with exposed negatively charged amino acid residues in the protein chain, modifying protein solubility. Additionally, basic amino acids can attach with aromatic amino acid residues through π-cation interactions, disrupting hydrogen bonds in the myosin backbone and partially preventing myosin aggregation. Lys can form complexes with metal ions, leading to the dissociation of actomyosin in proteins, thereby improving surimi gel strength. Glu and Asp can bind with amino acid residues like Ser, Thr, and Tyr on protein chains via ion–dipole interactions or interact with amino acid residues on myosin through hydrogen bonding during heating [80]. L-histidine, L-arginine, L-glutamine, and L-lysine have been research hotspots as additives for low-salt surimi gels [81,82,83]. Studies have reported that L-arginine (L-Arg) stabilizes proteins, prevents aggregation, and induces hydrophobic amino acid groups to engage with one another, enhancing the solubility of myofibrillar proteins. Compared to L-histidine, L-Arg has shown a more evident effect on the myofibrillar protein gel [84]. Additionally, the effect of cystine and L-lysine in low-salt surimi gels has also been described in [78]. Cystine is a weak oxidant; it causes the oxidation of SH groups on the surface of proteins, leading to the formation of S-S bonds [27], and lysine enhances the formation of cross-links mediated by endogenous transglutaminase that are established between lysine and glutamine amino acids. These amino acids improve the gel strength and thus the techno-functional properties of surimi gels by initially promoting protein denaturation or the unfolding of myofibrillar proteins, enabling the establishment of different types of bonds and resulting in compact and well-structured network gels. The combination of some of these amino acids with other components, such as oxidized caffeic acid, has also shown a strong potential for enhancing the gel properties of low-salt surimi gel [85].

#### 4.2.6. Addition of Proteins

The most frequently utilized proteins are egg white, whey protein concentrate, chicken or porcine plasma protein, etc. [86]. Nevertheless, due to an allergy to different animal proteins, mainly from cow’s milk, lactose intolerance problems, or religion, certain people cannot consume specific animal proteins. Therefore, plant proteins have gained relevance as effective additives to enhance surimi products’ gelation since they can interact with myofibrillar proteins through hydrophobic interactions and disulfide bonds, thus modifying the gel structure and functionality [19,87,88,89].

Concerning plant proteins, the most popular and reasonably priced plant proteins are soy isolate protein (SPI) [87], low-lectin bean protein (LLBP) [88], wheat gluten (WG) [89], and pea protein (PP) [18]. The effect of the incorporation of soy protein isolate has been reported to be effective in improving the mechanical properties in silver carp surimi gels in a relatively low concentration (6 g/100 g) [87]. The effect of the incorporation of LLBP in a range from 14 to 17 g/100 g in low-salt Alaska pollock surimi gels indicated a high correlation with the salt content. Better gels were achieved when the salt content was decreased, likely due to the presence of saline ions that modify the electrostatic equilibrium of the charges that stabilize the gel network in the gels with a reduced salt content [18]. WB and hydrolysed WB have also shown a positive effect on enhancing surimi gel properties [89]. The effect of these proteins has demonstrated their ability as gelation enhancers by enhancing conformational flexibility and structural stabilizing ability [86,90]. Although a limited number of existing studies have initially assessed the potential use of these plant proteins as gel enhancers in surimi gels within a range of less than 10 g/100 g, not all of them have been studied with a low salt content in the medium, and the mechanisms underpinning the reinforcement effects are still unclear, suggesting that the gel-enhancing effectiveness of plant proteins depends on their concentration and could be related to the presence of different salt levels in the system.

## 5. Techno-Functional Properties of Low-Salt Surimi Gels

Surimi-based products have a series of techno-functional properties, which depend fundamentally on the quality of the raw material (protein functionality and protein concentration), the additives incorporated, and the gelation process carried out. According to [15], the three most important functional characteristics in surimi-based products, related to or derived from the protein’s functionality, are colour, flavour, texture, and water holding capacity.

### 5.1. Texture

Texture is one of the most important sensory properties of surimi-based gels since it determines the degree of consumer acceptance.

Instrumental analysis is very useful for its study since there are several parameters that correlate well with its sensory analysis. One of the most used methods is the Puncture Test, which mimics the deformation that takes place during chewing of the food until the food breaks [55] and determines the breaking force (N) and breaking deformation (mm) (Figure 2).

Another method commonly used is the Texture Profile Analysis (TPA), which consists of a mechanical compression that aims to mimic the chewing process and determines the hardness (N), adhesiveness (N.s), springiness (mm), and cohesiveness [55].

**Figure 3:**
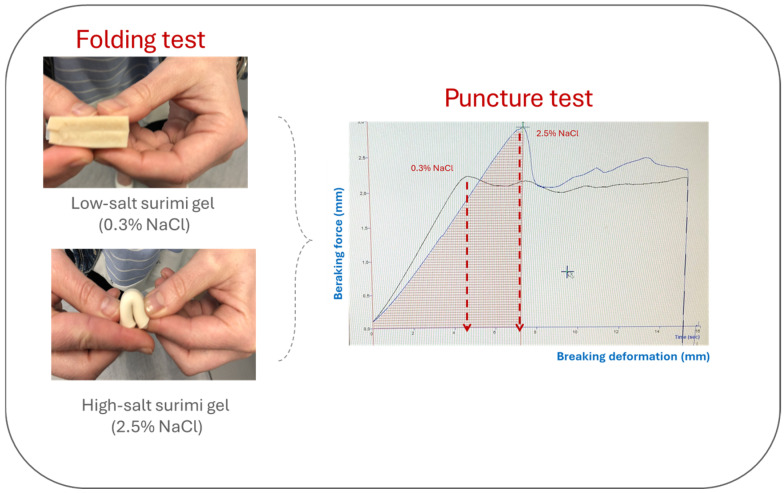
Effect of salt reduction on texture of surimi gels.

At reduced salt levels, the challenges in myosin unfolding and assembly often result in structural disorder and weak gels [82], which are typically reflected in a reduced gel strength (Figure 3). However, not all additives used as gelation enhancers for low-salt surimi gels have the same effect on the mechanical properties. Therefore, regulating the addition levels is essential to optimize the quality of surimi gels and prevent adverse effects on the formation of the gel network [22].

### 5.2. Water-Holding Capacity (WHC)

This is an essential characteristic of surimi gels that significantly affects their texture, mouthfeel, and overall quality. This property can be described as the ability of a food to retain its own moisture or added water (bound water, immobile water, and free water) when subjected to pressure, centrifugation, or heating forces [91]. These water molecules are firmly held within the gel matrix through hydrogen bonds and protein–water interactions [92]. The reduction in salt in surimi gels generally implies a reduction in the WHC, but the addition of different technological additives that act as gelation enhancers could increase the WHC. Depending on the additive, the enhancement is attributed to different processes: the exposure of hydrophilic groups, which bind more effectively with water molecules [93]; the increase in the contact area between protein and water molecules [87]; or the increase in the repulsion of protein groups due to the predominance of negative charges on the protein groups, which disorganizes the structure of the proteins and enhances the sites available to bind to water molecules [5].

### 5.3. Colour

Colour is one of the parameters that is modified during the gelation process due to protein aggregation. It is usually measured in the CIE L*a*b* colour space. In this space, colour is defined in a three-dimensional space, and each parameter corresponds to an axis, where L* indicates lightness (light to dark), a* is a colour coordinate from red to green, and b* is a colour coordinate from yellow to blue [15]. In surimi gels, special interest is usually paid to the brightness since it gives an idea of the degree of aggregation, and a lighter colour is associated with a higher quality. Due to protein aggregation, the amount of protein–protein bonds increases considerably, forming a compact protein structure that reflects light to a greater extent and therefore increases the luminosity [94]. According to this, all additives that enhance protein aggregation in low-salt surimi gels would also increase the lightness of the gel, which is not necessarily higher than the gels with a higher level of salt.

### 5.4. Flavour

The decrease in salt content in surimi-based products can considerably weaken their flavour, especially the perception of saltiness. The salty flavour of surimi gels has been related to the presence of adenosine triphosphate and its degradation products and to the formation of free amino acids, mainly alanine, a sweet amino acid, during thermal treatment [95,96]. In addition, it has been described that the addition of a small amount of yeast extract to reduced-salt surimi gels can enhance the salty and umami flavours of the product and, at the same time, reduce the sour and bitter flavours linked to the umami amino acids [80].

Achieving the optimal values of all of these parameters in low-salt surimi gels represents a major technological challenge for the surimi industry, which indicates that further research is required to achieve the desired properties.

## 6. Conclusions and Future Directions

In this paper, a comprehensive review of the most relevant low-salt surimi gelation enhancers has been provided, considering their feasibility for use by surimi product manufacturers or industries. Undoubtedly, salt reduction in surimi-based products poses a significant challenge for the industry, not only from technological and sensory perspectives but also in response to the growing consumer demand for healthier food options. In this context, surimi quality as a raw material is often poorly understood by consumers, who may question the nutritional value of this raw material and, by extension, the resulting surimi-based products. Therefore, it is essential to thoroughly describe the processing of this raw material and the importance of proper myofibrillar protein gelation to achieve products that are appealing to consumers. However, it is essential to inform consumers about the boundaries of surimi-based products by transmitting scientific information related to surimi processing, gelation, the effect of salt on the technological properties of the final products, and their nutritional value. To achieve this, education programs in schools, high schools, and universities, as well as well-designed marketing campaigns on TV, radio, and social media, would be effective options. As scientists, the best we can do is conduct research to provide society with reliable information and help disseminate it.

While surimi-based products typically require the addition of high levels of salt (1–3%) to obtain an adequate texture, such formulations may compromise their health benefits. In response, numerous countries and regions have introduced sodium-labelling regulations to indicate the level of salt in food products. Despite considerable efforts to develop low-salt surimi-based products, challenges remain, including issues with gel quality, increased costs, and a higher calorie content, all of which require further optimization. Moreover, it is important to recognize that both surimi-based and low-salt surimi-based products can be enriched with various ingredients (such as fibres, proteins, vitamins, and omega-3 fatty acids) that enhance their nutritional value and improve their final texture. Additionally, surimi-based products are fishery-derived, bone-free items that can serve as a valuable protein source for specific groups, such as children, young adults, and the elderly.

In this regard, substantial efforts must be directed toward the selection of surimi enhancers, either alone or in combination with different gelation methods (e.g., 3D printing, microwave processing, ultrasound, and high-pressure processing) that can enhance the physicochemical and sensory properties of low-salt surimi-based products. The most significant challenge for future research is the development of specific surimi gelation methods (additives and gelation techniques) that are affordable for the surimi-based products industry, which, together with consumers, remain the ultimate beneficiary of these advancements. Accordingly, the interaction between surimi production industries and researchers is fundamental to making progress in low-salt surimi gelation.

## Data Availability

No new data were created or analyzed in this study.
